# Personality and Its Partisan Political Correlates Predict U.S. State Differences in Covid-19 Policies and Mask Wearing Percentages

**DOI:** 10.3389/fpsyg.2021.729774

**Published:** 2021-09-27

**Authors:** Gene M. Heyman

**Affiliations:** Department of Psychology and Neuroscience, Boston College, Chestnut Hill, MA, United States

**Keywords:** Covid-19 restrictions, mask wearing, Big 5, *Conscientiousness*, *Openness*, political polarization, commonality regression, personality

## Abstract

A central feature of the Covid-19 pandemic is state differences. Some state Governors closed all but essential businesses, others did not. In some states, most of the population wore face coverings when in public; in other states, <50% wore face coverings. According to journalists, these differences were symptomatic of a politically polarized America. The Big 5 personality factors also cluster at the state level. For example, residents of Utah score high on *Conscientiousness* and low on *Neuroticism*, whereas residents of Massachusetts and Connecticut show the opposite pattern. In state-level regressions that controlled for partisan political allegiances, *Conscientiousness* was a significant (negative) predictor of the stringency of state Covid-19 restrictions, whereas *Openness* was a significant (positive) predictor of mask wearing. A number of the predictors were strongly correlated with each other. For example, the correlation coefficient linking *Openness* with the percentage of Democratic state legislators was *r* = 0.53. Commonality regression partitions the explained variance between the amount that is unique to each predictor and the amount that is shared among subsets of correlated predictors. This approach revealed that the common variance shared by *Conscientiousness, Openness* and partisan politics accounted for 34% of the state differences in Covid-19 policy and 35% of the state differences in mask wearing. The results reflect the importance of personality in how Americans have responded to the Covid-19 pandemic.

## Introduction

In response to the Covid-19 pandemic, the governors of California, New Mexico, and Maine mandated restaurants to stop serving patrons indoors, churches to close or greatly restrict attendance, and individuals to wear face coverings when in public. In contrast, the governors of South Dakota, Utah, and Kansas issued no such restrictions or quickly relaxed those that were in place and never mandated face coverings (Fernandez and Healy, 2020; Bunis and Rough, 2021). Journalists and social scientists attributed these differences to political and geographical ideological differences (Brenan, 2020; Pew Research Center, 2020; Gadarian et al., 2021). For instance, the Governors who issued the least restrictive guidelines were Republicans, whereas those who issued the most restrictive ones were typically, but not always, Democrats. However, there is more to political ideology than politics and geography. Psychologists and political scientists have shown that two of the Big 5 personality factors, *Conscientiousness* and *Openness*, reliably predict political ideology (e.g., Gerber et al., 2010) and cluster geographically in ways that fit the geographical and political clustering stressed in accounts of the U.S. response to Covid 19 (Rentfrow et al., 2009). States with populations that score high on *Conscientiousness* tend to vote for Republican presidential candidates, whereas states with populations that score high on *Openness* tend to vote for Democratic presidential candidates (see Supplementary Table 1). An article in *The New Yorker* on a small North Dakota town's struggle to come to a consensus on mask wearing nicely captured the role of individual differences (Gawande, 2021). Those pushing for issuing a mask mandate checked one or more of the following boxes: worked in health care, had a professional degree, was a progressive Democrat, or grew up out of state. Those against the proposal grew up in town or nearby, voted for Trump (some of whom had also voted for Barack Obama), and framed Covid-19 restriction in terms of local consequences, such as the threat to town businesses and the lost opportunities for high school athletes. For them, the Centers for Disease Control's (CDC) recommendations were worse than the disease. The debate was about how people led their lives as much as it was about health; it was deeply personal. (The labels for the personality factors are italicized to indicate that they identify patterns of responding on questionnaires and do not necessarily reflect ordinary word usage.).

The journalists' accounts are testable. In this report, I identify quantitative analogs for their observations and enter them into multiple regression models. My goal is to calculate how much of the variance in state-level responses to Covid-19 can be attributed to political orientation, to geography, and—in the spirit of the *New Yorker* article—to individual differences. The dependent measures are the stringency of state Covid-19 restrictions (Hallas et al., 2020) and the percentage of a state's population that wore masks when in public (Katz et al., 2020). The primary independent measures are *Conscientiousness* and *Openness* (John and Srivastava, 1999), each measured at the state level (Rentfrow et al., 2009), and the percentage of Democratic seats in state legislatures. Other predictors included the percentage of individuals who lived in a state that they were not born in, the percentage of the state residents who were not White-Non-Hispanics, and the percentage of the state population that lived in urban census tracts.

Notice that both the dependent and independent variables are at the state level. Some are directly so, such as the percentage of legislative seats held by Democrats, some are aggregated individual responses, as in the case of the personality measures. However, the observations that motivated this study include research on individuals as well as aggregated data, and the *Discussion* includes observations as to the possible relevance of the state-level findings reported here to individuals. This is an issue because it is possible for aggregated findings not to apply to individuals and vice versa (e.g., the “ecological fallacy,” Loney and Nagelkerke, 2014, Robinson, 1950). For example, individuals who score high on *Conscientiousness* tend to smoke less than individuals who score low on *Conscientiousness*, whereas there can be just the opposite correlation for states (e.g., see discussion by Wood and Rogers, 2011 and the example in Supplementary Table 1). However, the state-level and individual-level correlations between personality and partisan political ideology are similar so that it is reasonable to explore the state-level correlations on the basis of individual data. See the *Discussion* for further comments on this issue.

Previous research and logic suggest what to expect. Researchers consistently find that political orientation (Democrat or Republican) was a strong—if not the strongest—predictor of (1) what people have learned, (2) believe, (3) and feel about the pandemic (e.g., Allcott et al., 2020; de Bruin et al., 2020; Gadarian et al., 2021). For instance, in several studies, political partisanship was a stronger predictor of compliance with restrictions than was the likelihood of exposure to the virus (Gollwitzer et al., 2020; Makridis and Rothwell, 2020). Thus, given the previously mentioned correlations between personality and political ideology, logic suggests that *Openness* will be positively correlated with mask wearing and stricter restrictions on public behavior and that, conversely, *Conscientiousness* will be negatively correlated with mask wearing and stricter restrictions.

Although logical, the prediction runs counter to one of the most well-established findings in Big 5 research. In scores of studies, *Conscientiousness* predicted health promoting behaviors, such as higher exercise rates and lower obesity levels (e.g., Bogg and Roberts, 2004; Hampson et al., 2013), whereas, the correlations between *Openness* and health promoting behaviors were weak and in some cases negative (Terracciano and Costa, 2004; Zvolensky et al., 2015; Chapman et al., 2020). Thus, the health correlates of *Conscientiousness* and *Openness* suggest that states with populations that score higher on *Conscientiousness* will have stricter Covid-19 policies and a higher proportion of residents who wear masks; whereas the political correlates of *Conscientiousness* and *Openness*, say states with populations who score higher on *Openness* will take greater heed of the dangers of Covid-19.

However, two caveats are in order. First, these predictions are based on individual behavior, and, as discussed, may not apply to aggregated data. Second, for most Americans, the challenges imposed by Covid-19 are unprecedented, so that previous research may not provide a reliable guide. For instance, given that masks have not played a large role in American health practices, it is reasonable to suppose that *Openness*, which is correlated with the willingness to entertain new ideas (e.g., John and Srivastava, 1999), will prove more strongly correlated with mask wearing than will *Conscientiousness*. This turned out to be the case.

The primary goal of the present study, then, is to evaluate the degree to which *Conscientiousness* and *Openness* predict between-state variation in the response to the pandemic, using aggregated, state-level data, and controlling for partisan political allegiances, urbanization, race and other factors emphasized in popular accounts of state differences. To my knowledge, only one previous study has tested the role of personality on mask wearing while controlling for partisan political affiliations (Shook et al., 2020), but that study was completed prior to the April 3, 2020 CDC recommendation calling for mask wearing when in public, and no study has used personality to predict state Covid-19 policy. A secondary goal of this paper is methodological. I use “commonality analysis” as well as standard multiple regression techniques. Commonality analysis partitions the explained variance between those portions that are uniquely associated with the predictors and those portions that are shared among subsets of correlated predictors. When predictors are correlated, their shared variance with the dependent variable is implicit in the beta scores, so that including both approaches provides a more complete account of the data.

## Method

Journal articles, the U.S. Census Bureau, and university and private research organizations provided the data for this report. The units of analysis are the 50 U.S. states plus the District of Columbia (DC). The dependent measures are the stringency of state Covid-19 restrictions and the percentage of individuals in the states and DC who report wearing masks when out in public. The data are publicly available and de-identified; their use is exempted from review by the Boston College Institutional Review Board.

### Stringency

Oxford University's COVID-19 Government Response Tracker program, led by Hale et al. (2021) of the Blavatnik School of Government, created the state stringency rankings for the 50 U.S. states and DC (Hallas et al., 2020). The research team included more than 50 individuals who gathered and coded publicly available reports on the Covid-19 pandemic. Four indictors make up the stringency scale: (1) a containment and health index, which combines restrictions and closures with health measures, such as testing policy and contact tracing, (2) economic policies, such as the level of funding for recently unemployed workers, (3) a stringency index, which records the strictness of the policies that restrict public behavior, and (4) an index that tracts the overall government approach to lockdown policies. The research team translated the four indices into an additive “stringency” scale of 1–100, with 100 defined as the most “stringent.” The scale used in the present report reflects data collected from late August to early December of 2020.

### Mask Wearing

The New York Times enlisted Dynata, a survey firm, to conduct an online, nation-wide, county-level survey of mask wearing (Katz et al., 2020; New York Times, 2021). The survey posted the question: “How often do you wear a mask in public when you expect to be within six feet of another person?” Respondents selected one of five answers, ranging from “never” to “always.” The Times reported that there were 250,000 responses between July 2 and July 14 of 2020. To obtain state-level results, I weighted the survey's finding by each county's share of the state population and then summed across counties (U.S. Census Bureau, Population Division, 2020; New York Times, 2021) and selected the “always” answer as the dependent variable.

### Personality

Rentfrow et al.'s 2008 summary of a nation-wide, 44 question Big-5 personality survey (John and Srivastava, 1999; Rentfrow et al., 2008) provided the state-level *Conscientiousness* and *Openness* measures. The survey recruited 619,397 participants and was carried out between December 1999 and January 2005. The researchers aggregated the results by state and then ranked each state on each of the Big 5 dimensions. The percentage of respondents from each state was proportional to the state's population (*r* = 0.98), and the race and ethnicity of the participants were roughly proportional to the race and ethnicity makeup of each state. The average inter-item alpha reliabilities for each of the five personality measures were 0.81 for individuals and 0.89 for states. The test-retest state-level scores were similar, with correlation coefficients that ranged from 0.77 to 0.88. For *Openness* and *Conscientiousness*, the test-retest correlations were both 0.88. Importantly, the factor structure for the aggregated state data matched that for individuals, selected independently of residence.

A central feature of *Openness* is interest in new ideas and experiences. In keeping with this observation, individuals who score high on *Openness* tend to favor same-sex marriage, support government health care programs, and dislike conventional music (Rentfrow and Gosling, 2003; Gerber et al., 2010). In contrast, one of the facets of *Conscientiousness* is adherence to norms; in keeping with this observation, individuals who score high on *Conscientiousness* are less likely to favor same-sex marriage, less likely to support a greater role for government in health care, and prefer conventional music (Rentfrow and Gosling, 2003; Gerber et al., 2010). Put more generally, the success of the Big 5 personality inventory reflects the everyday experience that individuals often behave, think and feel in similar ways under different situations.

Notice that the personality data were collected over the years 1999 to 2005, whereas the Covid-19 responses took place in 2020–2021. To check whether the personality factors remained relevant to current events pertinent to this study, I evaluated the state-level correlations between percentage of votes for Democratic presidential candidates and *Conscientiousness* and *Openness* for the years 1996 to 2020 (e.g., “1996 United States Presidential Election,” 2021). Supplementary Table 1 shows the results. The correlations between the two personality measures and partisan political preferences were significant in each election and did not reveal any clear temporal trends. For example, the correlations for 2020 (*rs* = −0.34 and 0.58) were about the same or higher than for 2000 (*rs* = −0.35 and 0.47). Thus, the assumption that the personality scores reflect current state populations has empirical support.

### Political Partisanship

There were several possible measures of political preferences: the percentage of presidential votes for Democratic and Republican candidates, the political party of the state governor, and the partisan makeup of state legislatures. On the basis of its geographic structure and the sheer numbers of elected officials (12,794), I selected the partisan makeup of state legislatures to represent the average resident's political orientation. The National Conference of State Legislatures (2019) provided the data.

### Urbanization

The Census Bureau provided the data on urbanization. Its classification system is based on population density. The state data were downloaded from Iowa State University Community Indicator website (Iowa State University, Iowa Community Indicator Programs, 1995–2021).

### Race and Ethnicity

In the U.S., Covid-19 cases and deaths vary according to race and ethnicity (e.g., Webb Hooper et al., 2020; Centers for Disease Control and Prevention, 2021). States differ in terms of the percentages of White Non-Hispanic residents. Accordingly, the proportion of individuals in each state who were not Non-Hispanic Whites was included as a predictor. They are referred to as “Non-Whites.” The U.S. Census Bureau's American Community Survey five year estimates (2015–2019), Table B03002, provided these data (U.S. Census Bureau, 2021a).

### Income

To estimate the influence of income, I used the 2018 median state income adjusted for cost of living. The Census Bureau's Current Community Survey provided the income estimates, and the *Council for Community and Economic Research* provided the cost of living estimates. The *Advisors Perspectives* website published the data (Mislinski, 2019).

### Education

The index for state differences in education level was the U.S. Census Bureau's estimate of the percentage of residents in each state who earned a college degree by age 25 (U.S. Census Bureau, 2021b). The survey is a five-year estimate for the years 2013–2017.

### Percent of State Population Not Born In-State

The *New Yorker* story on a North Dakota town's struggle to agree on issuing a mask mandate suggested that individuals who had moved from rural to urban areas or who had moved into a rural area from out of state were more likely to view the response to Covid-19 primarily in terms of its health benefits, whereas people in rural areas who had not moved away were more likely to consider the response to Covid-19 in terms of its economic, social, and psychological impact on their families and neighbors. These observations suggested that the percentage of state residents born out of state might prove an informative predictor of state Covid-19 policy and the likelihood of mask wearing. Conveniently, the Census Bureau tracks these numbers in their “State of Residence by State of Birth” tables. I used the tables for 2019 (U.S. Census Bureau, 2020).

### Cumulative Cases

Johns Hopkins University provides a regularly updated count of cases by state. I selected the data that were updated February 19, 2021. They were published by CNN (2021).

### Statistical Analyses

Multiple linear regression and commonality regression were used to evaluate the relationship between the predictors, stringency, and mask wearing. The models always included *Openness* and *Conscientiousness*. Thus, the regressions tested the degree to which personality explained state differences in the response to the pandemic, while controlling for the predictors stressed in previous accounts. To keep the ratio of observations to predictors above the recommended ten to one ratio, the primary analyses limited the number of predictors to four. This was arranged by using Stata's *tryem* command. This is a “brute force” method for finding the subset of *k* predictors that accounts for the most variance. For instance, to find the four predictors that explains the most variance in the dependent measure, *tryem* evaluates every possible subset of four predictors. In addition, to ensure that *tryem'*s choice of four predictors was robust, I also evaluated whether the beta scores varied as function of regressions that included more than the two strongest non-personality predictors. Supplementary Tables 2, 3 list the results.

Commonality regression partitions the explained variance between the portions that are unique to each predictor and the portions associated with the common variance of the correlated predictors. Although shared (rather than unique) variance among the predictors may account for much of the variance in the dependent variable, the beta scores do not make this explicit. Thus, commonality regression can provide a more complete account of the relations between the independent and dependent variables than do beta scores alone (e.g., Zientek and Thompson, 2006; Nimon and Oswald, 2013). Kim Nimon generously provided the SPSS syntax file that executed the commonality regressions (personal communication). Other statistical analyses were conducted with Stata 16.

Notice that the regressions include the entire population of states; inferential statistics are included but are not paramount. Rather, proportions of variance accounted for (referred to as VAF) provide the most relevant statistical results.

## Results

Table 1 lists the averages, standard deviations, and medians for the variables used in the analyses. For most variables, the means and medians were about the same. In support of this point, most skewness values were <0.50 and only one (percent of state population that graduated college), was >1.0. The Shapiro-Francia test was used to evaluate normality (Royston, 1993). The null hypothesis is that the distribution is normal. Both dependent measures yielded non-significant z-scores, as did seven of the nine predictors. The z-scores for the percentage of state population that graduated college and Covid-19 case rates were statistically significant. However, the Shapiro-Francia tests were no longer significant when outliers were removed. Maine and Vermont had uniquely low Covid-19 case rates, and the District of Columbia had a uniquely high college graduation rate. Nevertheless, these states were retained for all analyses as removing them had very little influence on the magnitudes of the regressions and correlations. For instance, correlations between the two dependent variables and Covid-19 case rates were statistically significant with and without the inclusion of Maine and Vermont; similarly, the correlations with the two dependent variables and college graduation remained insignificant with and without the District of Columbia—as shown next in Table 2.

**Table 1:** Descriptive statistics used in the analyses.

**Variable**	**Average**	**Stand. dev**.	**Median**
State stringency rank	26	14.87	26
% of state population who wear mask in public	59.9	13.6	61.1
State Covid-19 cases/100 k Residents	8,402.3	2,408.7	8,790.5
State *Conscientiousness* rank	26	14.87	26
State *Openness* rank	26	14.87	26
% of state legislators who are Democrats	47.7	20.50	43.7
% of state population who live in urban census tract	74.1	14.9	74.2
% of state population who are Non-White	31.9	16.2	28.4
% of state population who graduated college	30.6	6.2	29.9
% of state population born out of state	43.1	11.6	40.9
State median household purchasing power ($)	62,000	9,659	63,200

**Table 2:** Bivariate correlations.

	**Covid-19** ** restrictions**	**% Always** ** wear mask**	**Cases/** **100 k**	**Conscientiousness**	**Openness**	**% Democratic** ** legislature**	**Urbanization**	**% Non-white**	**% Born out of state**	**Median** ** income** ** purchasing power**	**% College** ** graduate**
Covid-19 restrictions	1										
% Always wear mask	0.70^***^	1									
Cases/100 k	−0.55^***^	−0.50^***^	1								
Conscientiousness	−0.50^***^	−0.28^*^	0.45^**^	1							
Openness	0.41^**^	0.58^***^	−0.37^*^	−0.12	1						
% Democratic legislature	0.77^***^	0.83^***^	−0.58^***^	−0.44^**^	0.53^**^	1					
Urbanization	0.29^*^	0.63^***^	0.004	0.02	0.45^**^	0.51^***^	1				
% Non-white	0.31^*^	0.60^***^	−0.11	0.06	0.25	0.49^**^	0.65^***^	1			
% Born out of state	0.21	0.38^*^	−0.26	−0.17	0.41^**^	0.36^*^	0.34^*^	0.32^*^	1		
Median income purchase power	−0.29^*^	−0.26	0.13	0.20	−0.11	−0.33^*^	−0.18	−0.51^***^	−0.12	1	
% College graduate	0.17	0.21	−0.20	−0.17	0.36^*^	0.28^*^	0.32^*^	0.21	0.33^*^	−0.21	1

* *≤ 0.05;*

** *≤ 0.005;*

*** *≤ 0.0005*.

Table 2 lists the correlations between the stringency of state Covid-19 restrictions, the percentage of individuals in each state who wore a face covering when in public, and the factors which have figured most prominently in accounts of state-level differences in the response to the pandemic.

By conventional standards (e.g., Cohen, 1992), the majority of the correlations ranged from “medium” to “large.” The correlations linking the percentages of elected Democrats in state legislatures to mask wearing and stringency were *r* = 0.83 and *r* = 0.77, respectively. *Conscientiousness* varied inversely with the percentage of state legislative seats held by Democrats (*r* = −0.44), whereas *Openness* varied directly with Democratic state legislators (*r* = 0.53). Taken together, these correlations predict that the two personality measures will be moderately to strongly correlated with stringency and mask wearing. As expected, the correlations linking *Conscientiousness* with stringency and mask wearing were *r* = −0.50 and *r* = −0.28, respectively, and the correlations linking *Openness* to these two Covid-19 responses were *r* = 0.41 and *r* = 0.58, respectively. Thus, personality scores, the percentage of Democrats elected to state office, the stringency of state ordered restrictions, and percentage of state residents who always donned a face covering when in public rose and fell together. The non-political and non-personality predictors (e.g., income and educational attainment) typically had moderate correlations with the other variables—with the exception of urbanization and the percentages of each state's Non-White residents. These two predictors were highly correlated with each other (*r* = 0.65), and both were strongly correlated with mask wearing, *Openness*, and the percentage of state offices held by Democrats. In sum, there are two types of states: those with large urban and Non-White populations, who vote for Democrats, score high on *Openness*, and wear masks when in public; and those with large rural and mostly White, populations, who vote for Republicans, score high on *Conscientiousness*, and are less likely to wear masks when in public. In addition, Table 2 shows that *Openness* and *Conscientiousness* made opposite predictions, although the negative correlation linking the two personality factors was not particularly large (*r* = −0.12).

Table 3A lists the multiple regression results for the degree of stringency in state mandated restrictions. The predictors were *Conscientiousness* and *Openness* and the two non-personality factors which when combined with the personality measures explained the most variance in stringency. Put another way, the regression tested whether personality contributes to state differences while including the two most powerful non-personality predictors. The partisan makeup of state legislatures and percentage of state residents born out of state were the two strongest non-personality predictors and *Conscientiousness* was the strongest personality predictor. Notice that its sign is negative, meaning states with higher *Conscientiousness* scores had laxer restrictions. Together, these four predictors accounted for 63% of the variance in the rank order stringency of state Covid-19 restrictions.

**Table 3A:** State *Conscientiousness* rank and political partisanship predict rank-order stringency of state Covid-19 restrictions.

**Dependent variable:** **stringency of state Covid-19 restrictions**	**Coefficient**	**Std. Error**	***t***	***P* > |*t*|**	**Beta**
State *Conscientiousness* Rank	−0.213	0.101	−2.11	0.040	−0.213
State *Openness* Rank	0.061	0.111	0.55	0.583	0.061
% Democrats in state legislature	49.08	8.62	5.70	0.000	0.677
Percent born in another state	−0.121	0.128	−0.95	0.348	−0.094
Constant	11.74	6.815	1.72	0.092	

*N = 51, R^2^ = 0.63, Adjusted R^2^ = 0.60, F_(4,46)_ = 19.89, Prob > F = 0.000*.

*Heteroscedasticity test (Breusch-Pagan/Cook-Weisberg): X^2^ < 0.005, p = 0.956.*

*VIF = 1.46 (1.24 – 1.77)*.

The bottom row of the table includes two widely used regression diagnostic tests. The Breusch and Pagan (1979)/Cook and Weisberg (1983) test shows that the residuals met the homoscedasticity assumption (approximately equal variances). The variance inflation test reveals that collinearity had little influence on the regression results. A Cook's distance test was run to check whether one or more states had a disproportionate influence on the regression results. The largest Cook's distance value was 0.22. Removing this state (New Mexico) from the analyses increased the variance accounted for percentage by a little over 3% (to 67%) and the magnitudes of the *Conscientiousness* and *Openness* coefficients (from −0.21 to −0.31 and from 0.06 to 0.09). However, New Mexico was retained in all analyses, as removing it did not alter significance levels for any of the predictors.

Table 3B summarizes the multiple regression results for the percentage of individuals in each state who report they always wear a face covering when in public. The strongest two non-personality predictors for all possible sets of four predictors that included *Conscientiousness* and *Openness* were the partisan makeup of the state legislatures and the percentage of Non-White state residents. Together they accounted for 76% of the variance in mask wearing. The percentage of Democratic seats in the legislature was again the strongest non-personality predictor, and for mask wearing, *Openness* was the strongest personality predictor.

**Table 3B:** State *Openness* rank, partisan politics, and % of Non-White state residents predict % of state population who always wear masks in pubic.

**Dependent variable:** **% of state residents who always wear mask in public**	**Coefficient**	**Std. Error**	***t***	***P* > |t|**	**Beta**
State *Conscientiousness* Rank	−0.0001	0.0008	−0.15	0.882	−0.013
State *Openness* rank	0.0018	0.0008	2.31	0.025	0.200
% Democrats in state legislature	0.3889	0.0748	5.20	0.000	0.587
% Not white state residents	0.2205	0.0746	2.95	0.005	0.262
Constant	0.2985	0.0393	7.60	0.000	

*N = 51, R^2^ = 0.760, Adjusted R^2^ = 0.739, F_(4,46)_ = 36.46, Prob > F = 0.000*

*Heteroscedasticity test (Breusch-Pagan/Cook-Weisberg): X^2^ = 1.26, p = 0.261*.

*VIF = 1.71 (1.43 – 2.44)*.

As in Table 3A, the bottom row statistics reveal that the residuals met the homoscedasticity assumption, and the degree of collinearity was well below the level for concern regarding the reliability of the regression coefficients. The largest Cook's distance value was 0.31. Removing this case from the analysis (District of Columbia) increased the variance accounted for from 76.0 to 79.6%. However, as there were no changes in significance level, the District of Columbia was retained in all analyses.

As an additional test of the robustness of the regression beta scores, I modified the regression context by also including the predictors that were not included in Tables 3A,B. For example, the beta coefficients for regressions that included the predictors in Tables 3A,B were compared with those for regressions that included these four plus the remaining five predictors listed in Tables 1, 2. Supplementary Tables 2, 3 list the results. For the stringency regressions, the average absolute median change in the value of the four coefficients in Table 3A was 0.017 and the rank order remained the same regardless of context. For the mask wearing regressions, the absolute median change in the value of the four coefficient in Table 3B was larger, 0.048, and, as with stringency, their rank order remained the same regardless of context. Thus, the regression coefficients were reasonably stable.

**Figure 1:**
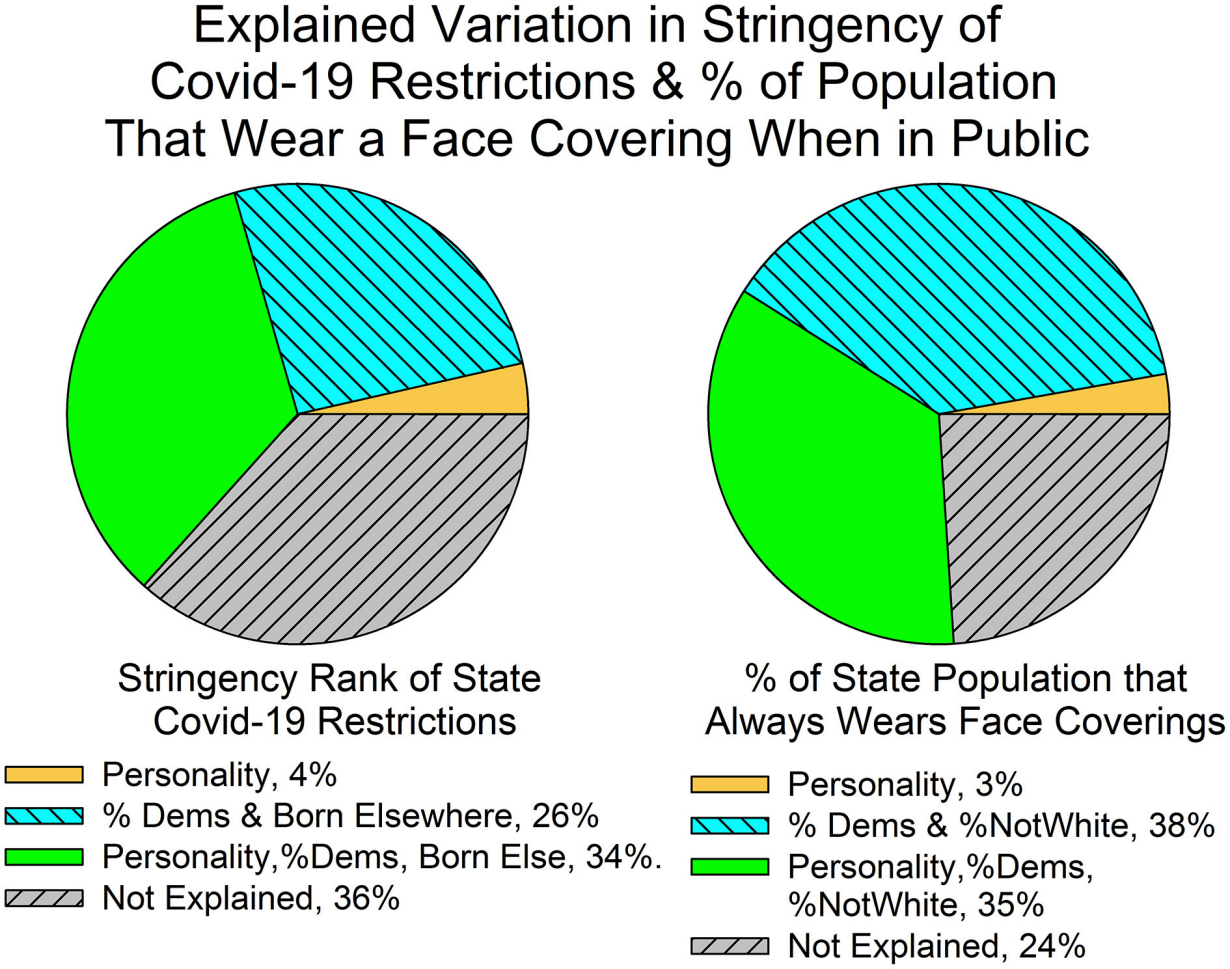
The sectors represent the common variance of the two personality factors, the two non-personality factors, and the shared variance among the two personality and the two non-personality measures. The size of the sector is proportional to the amount of variance its predictors explain. See text for other details.

When the predictors are correlated with one another, commonality regression provides information not apparent in the beta scores. Tables 4A,B and Figure 1 summarize these results. The tables' second rows list the explained variance that is unique to each of the four predictors identified in the tables' top rows, and the third rows list the explained variance credited to the subsets that include the predictors identified in the top rows. For instance, the third-row entry for *Conscientiousness* in Table 4A is the correlation between stringency and the common variance shared by *Conscientiousness*, percentage of Democratic seats in the state legislatures, and percentage of state residents born out of state. Notice that it is possible for a given predictor to be a member of more than one subset. This implies that the VAF subset commonality scores can add up to more than 100%. (They would sum to zero if the predictors were perfectly independent of one another.)

**Table 4A:** Commonality regression analysis of the stringency rank of state Covid-19 restrictions.

**Predictor/predictor subsets**	***Conscientiousness*** **Rank**	***Openness*** **Rank**	**% Democratic** **state legislators**	**% Born out** **of state**
% Variance accounted for: Unique to the predictor	3.6	0.24	25.8	0.7
% Variance accounted for common to subsets that include the predictor	21.76	16.1	33.4	3.5

**Table 4B:** Commonality regression analysis of the percentage of state population who always wear a face covering when in public.

**Predictor/predictor subsets**	***Conscientiousness*** **Rank**	***Openness*** **Rank**	**% Democratic** **state legislators**	**% Non-white**
% Variance accounted for: Unique to the predictor	0.0001	2.8	14.1	4.6
% Variance accounted for common to subsets that include the predictor	7.7	30.5	54.1	31.3

*See text for further details*.

The left-side pie chart in Figure 1 summarizes the commonality analysis of state stringency rankings. The sectors are proportional to the explained variance for personality (*Conscientiousness, Openness*, and the two together), the two non-personality variables (political partisanship, the percentage of state residents born out of state, and the two together), and the common variance that emerged as a function of the correlations between the four predictors. The legend provides the numerical percentage scores for each sector. These are mutually exclusive categories so that they sum to the total explained variance (63.4%). About half of the explained variance was due to the predictors' unique association with stringency and about half was due to the personality plus non-personality subsets.

The right-side pie chart in Figure 1 summarizes the commonality regression analysis of mask wearing. The pattern of results are similar to those for stringency. Again, about half of the explained variance was due to the overlap between mask wearing and the subsets that included one or both personality measures and one or both non-personality measures. Thus, as with stringency, personality's predictive influence on mask wearing is embedded in its correlations with the non-personality predictors.

## Discussion

State *Conscientiousness* rank, the percentage of Democratic seats in the state legislature, and the percentage of individuals who were born out of state accounted for 63% of the between state variance in Covid-19 policies. State *Openness* rank, the percentage of Democratic seats in the state legislature, and the percentage Non-Whites accounted for 76% of the between state variance in mask wearing. Thus, the measures used in this report provided a strong account of the much-discussed state differences in the response to Covid-19.

As noted in the *Introduction*, the correlates of *Conscientiousness* and *Openness* predicted conflicting outcomes for the state analyses presented here. The political correlates predicted that states that ranked high on *Openness* would have more severe restrictions and a higher percentage of mask wearing; in contrast, the health correlates predicted that the residents of states that ranked high on *Conscientiousness* would take a more cautious approach to the pandemic. The political correlates predicted the results. This is consistent with other findings at both the individual and state level, as briefly described below. But, regardless of supporting evidence, the data presented here do not eliminate the problem that extrapolations from aggregate to individual data can prove false. This needs to be addressed first.

Assume the following three relations: (1) the likelihood of donning a protective face covering is a negative function of *Openness* [which according to many studies is a reasonable possibility (e.g., Bogg and Roberts, 2004)], but a positive function of the tendency to vote for Democratic legislative candidates (as observed); (2) U.S. State A is heavily populated by individuals who vote Republican and score low on *Openness;* (3) and U.S. State B is heavily populated by individuals who vote for Democrats and score high on *Openness*. Under these conditions, it is possible for State B to have a higher rate of mask wearing and a higher *Openness* rank, thereby yielding a positive correlation between *Openness* and mask wearing at the state level. Yet, Condition 1 (above) says that for individuals there is a negative relationship between *Openness* and mask wearing. Indeed, if we measured the relationship between *Openness* and mask wearing within State A and within State B, we would find that individuals who scored higher on *Openness* were less likely to wear masks (just the opposite of the state-level correlation). Hence, it is logically possible for state-level correlations to move in just the opposite direction of individual-level correlations.

However, the following considerations predict that a replication of the present study with individuals would yield the state-level pattern of findings reported in the tables and figures of this report, the ecological fallacy notwithstanding. First, the direction of the correlations between personality and political orientation are the same at the state level and individual level (e.g., Rentfrow et al., 2009; Gerber et al., 2010), in contrast to the hypothetical example just given. Second, a common theme in Covid-19 studies is that political partisanship is the strongest predictor of what individuals know, believe, and do in regards to the threats posed by Covid-19 (e.g., Allcott et al., 2020). Similarly, partisan political orientation was the strongest state-level predictor. Thus, given the strength of the correlation between personality and political orientation at the individual and state level, the strength of the relationship between political orientation and the response to Covid-19 at the individual and state level, and that the individual and state correlations are in the same direction, the most reasonable expectation is that the state-level findings reported here correctly predict individual results. The state-level personality results are also in line with individual-level aspects of *Openness* emphasized by Götz et al. (2020) in their account of sheltering in place. They point out that *Openness* predicts “more accurate risk perceptions” and the “sense that we are all in this together.” To this, we can add that *Openness* predicts a willingness to entertain novel practices, whereas *Conscientiousness* is correlated with adherence to norms. Thus, when confronted with an unprecedented event, it is reasonable to expect that individuals who score high on *Openness* will be more likely than those who score high on *Conscientiousness* to take unprecedented actions, such as wearing a face covering when in public. However, the summary presented here is not a proof. Rather, it is the simplest, most consistent summary of the data—and an invitation to conduct an individual-level replication of the present study.

The commonality regression analysis revealed several interesting trends. First, about half of the explained variance was due to the subsets of correlated predictors and about half was due to the unique contribution of each predictor. Second, partisan politics accounted for the most unique and common variance. Third, the ratio of unique to common explained variance varied across predictors: for all save the makeup of the state legislature, it greatly favored common variance, which is to say, the correlated predictor subsets. As there are no other commonality analyses of the response to Covid-19, the generality of the present results remains unknown.

## Limitations, Contributions

There are several limitations. First, as emphasized throughout, this study is at the state level so that its implications for individual-level correlations are not definitive. That said, it is also the case that there are advantages to using aggregated data. The sample sizes are huge, and the error involved with individual variation averages out. Thus, aggregated personality data may reveal relations that are present but hard to detect with individual data. Second, this study provided no analysis of Covid-19 case rates or death rates. These analyses require considerations that are beyond the scope of this paper, such as information regarding pre-existing medical conditions and the age of those infected. Third, the analyses do not shed light on the interesting question of the nature of the relationship between personality and political ideology. Given, the strong correlations between personality, partisan political preferences, and mask wearing (Table 2), this topic deserves much attention.

## Conclusions

*Conscientiousness* and *Openness* predicted state differences in Covid-19 policy and mask wearing in multiple regression analyses that included controls for political partisanship. The commonality analysis revealed that shared variance among the correlated predictors was a major predictor of state differences in Covid-19 policy and mask wearing. Gawande titled his *New Yorker* article: “Don't tell me what to do.” The analyses reported here suggest a modification of this title: “Don't tell me who to be.”

## Data Availability Statement

The original contributions presented in the study are included in the article/Supplementary Material, further inquiries can be directed to the corresponding author/s.

## Ethics Statement

Ethical approval was not provided for this study on human participants because study uses publicly available, de-identified, state-level data that is not reviewed by our review board. Written informed consent for participation was not required for this study in accordance with the national legislation and the institutional requirements.

## Author's Note

Covid-19 case rates are significantly lower in U.S. states that mandated stricter Covid-19 restrictions. According to most observers, the state differences reflect political (e.g., Democratic and Republican) and geographical (e.g., rural and urban) ideological differences. But, in the United States, political ideology and personality—as measured by the “Big 5” procedure—go hand in hand. In this report, multiple regression analyses that included controls for political affiliation, race and geography revealed that state-level Big 5 personality differences predicted state differences in the stringency of Covid-19 restrictions and the likelihood of mask wearing. These results suggest that the strong correlations between partisan political affiliation and the response to Covid-19 in the U.S. are in part due to the strong correlations between personality and political ideology.

## Author Contributions

The author confirms being the sole contributor of this work and has approved it for publication.

## Conflict of Interest

The author declares that the research was conducted in the absence of any commercial or financial relationships that could be construed as a potential conflict of interest.

## Publisher's Note

All claims expressed in this article are solely those of the authors and do not necessarily represent those of their affiliated organizations, or those of the publisher, the editors and the reviewers. Any product that may be evaluated in this article, or claim that may be made by its manufacturer, is not guaranteed or endorsed by the publisher.

## References

[B1] Allcott H. Boxell L. Conway J. Gentzkow M. Thaler M. Yang D. (2020). Polarization and public health: partisan differences in social distancing during the coronavirus pandemic. J. Public Econ. 191:104254. 10.1016/j.jpubeco.2020.104254 32836504 PMC7409721

[B2] Bogg T. Roberts B. W. (2004). Conscientiousness and health-related behaviors: a meta-analysis of the leading behavioral contributors to mortality. Psychol. Bull. 130:887. 10.1037/0033-2909.130.6.887 15535742

[B3] Brenan M. (2020, July 13). Americans' face mask usage varies greatly by demographics. *Gallup*. Available online at: https://news.gallup.com/poll/315590/americans-face-mask-usage-varies-greatly-demographics.aspx (accessed March 11, 2021).

[B4] Breusch T. S. Pagan A. R. (1979). A simple test for heteroscedasticity and random coefficient variation. Econometrica 47, 1287–1294. 10.2307/1911963

[B5] Bunis D. Rough J. (2021). State by State Coronavirus-Related Restrictions. AARP. Available online at: https://www.aarp.org/politics-society/government-elections/info-2020/coronavirus-state-restrictions.html (accessed March 13, 2021).

[B6] Centers for Disease Control Prevention (2021). Health Equity Considerations and Racial and Ethnic Minority Groups. Available online at: https://www.cdc.gov/coronavirus/2019-ncov/community/health-equity/race-ethnicity.html (accessed April 19, 2021).

[B7] Chapman B. P. Elliot A. Sutin A. Terraciano A. Zelinski E. Schaie W.. (2020). Mortality risk associated with personality facets of the Big Five and interpersonal circumplex across three aging cohorts. Psychosom. Med. 82, 64–73. 10.1097/PSY.0000000000000756 31688676

[B8] CNN (2021). Tracking Covid-19 Cases in the US. Available online at: https://edition.cnn.com/interactive/2020/health/coronavirus-us-maps-and-cases/ (accessed February 22, 2021).

[B9] Cohen J. (1992). A power primer. Psychol. Bull. 112, 155–159. 10.1037/0033-2909.112.1.155 19565683

[B10] Cook R. D. Weisberg S. (1983). Diagnostics for heteroscedasticity in regression. Biometrika 70, 1–10. 10.1093/biomet/70.1.1

[B11] de Bruin W. B. Saw H. W. Goldman D. P. (2020). Political polarization in US residents' COVID-19 risk perceptions, policy preferences, and protective behaviors. J. Risk Uncertain. 61, 177–194. 10.1007/s11166-020-09336-3 33223612 PMC7672261

[B12] Fernandez M. Healy J. (2020, November 21). 1 America, 1 pandemic, 2 realities. The New York Times. Available online at: https://www.nytimes.com/2020/11/21/us/coronavirus-south-dakota-new-mexico.html (accessed January 25, 2021).

[B13] Gadarian S. K. Goodman S. W. Pepinsky T. B. (2021). Partisanship, health behavior, and policy attitudes in the early stages of the COVID-19 pandemic. PLoS ONE 16:e0249596. 10.1371/journal.pone.0249596 33826646 PMC8026027

[B14] Gawande A. (2021, February 8). Inside the Worst-Hit County in the Worst-Hit State in the Worst-Hit Country. *The New Yorker*. Available online at: https://www.newyorker.com/magazine/2021/02/15/inside-the-worst-hit-county-in-the-worst-hit-state-in-the-worst-hit-country (accessed May 25, 2021).

[B15] Gerber A. S. Huber G. A. Doherty D. Dowling C. M. Ha S. E. (2010). Personality and political attitudes: relationships across issue domains and political contexts. Am. Polit. Sci. Rev. 104, 111–133. 10.1017/S0003055410000031

[B16] Gollwitzer A. Martel C. Brady W. J. Pärnamets P. Freedman I. G. Knowles E. D.. (2020). Partisan differences in physical distancing are linked to health outcomes during the COVID-19 pandemic. Nat. Hum. Behav. 4, 1186–1197. 10.1038/s41562-020-00977-7 33139897

[B17] Götz F. M. Gvirtz A. Galinsky A. D. Jachimowicz J. M. (2020). How personality and policy predict pandemic behavior: understanding sheltering-in-place in 55 countries at the onset of COVID-19. Am. Psychol. 76, 39–49. 10.1037/amp0000740 33475389

[B18] Hale T. Angrist N. Goldszmidt R. Kira B. Petherick A. Phillips T.. (2021). A global panel database of pandemic policies (Oxford COVID-19 Government Response Tracker). Nat. Hum. Behav. 5, 529–538. 10.1038/s41562-021-01079-8 33686204

[B19] Hallas L. Hatibie A. Majumdar S. Pyarali M. Hale T. (2020). “Variation in US States' Responses to COVID-19 2.0.” Blavatnik School of Government Working Paper. Available online at: www.bsg.ox.ac.uk/covidtracker

[B20] Hampson S. E. Edmonds G. W. Goldberg L. R. Dubanoski J. P. Hillier T. A. (2013). Childhood conscientiousness relates to objectively measured adult physical health four decades later. Health Psychol. 32, 925–928. 10.1037/a0031655 23527514 PMC3754851

[B21] Iowa State University, Iowa Community Indicator Programs (1995–2021). Urban Percentage of the Population for States, Historical. Available online at: https://www.icip.iastate.edu/tables/population/urban-pct-states (accessed February 1, 2021).

[B22] John O. P. Srivastava S. (1999). The Big-Five Trait Taxonomy: History, Measurement, and Theoretical Perspectives. Vol. 2. Berkeley: University of California, 102–138.

[B23] Katz J. Sanger-Katz M. Quealy K. (2020, July 17). A detailed map of who is wearing masks in the U.S. The New York Times. Available online at: https://www.nytimes.com/interactive/2020/07/17/upshot/coronavirus-face-mask-map.html

[B24] Loney T. Nagelkerke N. J. (2014). The individualistic fallacy, ecological studies and instrumental variables: a causal interpretation. Emerg. Themes Epidemiol. 11:18. 10.1186/1742-7622-11-18 25745504 PMC4350299

[B25] Makridis C. Rothwell J. T. (2020). The Real Cost of Political Polarization: Evidence From the COVID-19 pandemic. SSRN 3638373.

[B26] Mislinski J. (2019, December 19). Median household purchasing power for the 50 states and DC. Advisor Perspectives. Available online at: https://www.advisorperspectives.com/dshort/updates/2019/12/19/median-household-purchasing-power-for-the-50-states-and-dc (accessed January 17, 2021).

[B27] National Conference of State Legislatures (2019). 2019 State & Legislative Partisan Composition. Available online at: https://www.ncsl.org/Portals/1/Documents/Elections/Legis_Control_2019_February%201st.pdf (accessed December 29, 2020).

[B28] New York Times (2021). Coronavirus (Covid-19) Data in the United States. Available online at: https://github.com/nytimes/covid-19-data (accessed March 1, 2021).

[B29] Nimon K. F. Oswald F. L. (2013). Understanding the results of multiple linear regression: beyond standardized regression coefficients. Org. Res. Methods 16, 650–674. 10.1177/1094428113493929

[B30] Pew Research Center (2020, June 25). Republicans, democrats move even further apart in coronavirus concerns. Pew Research Center. Available online at: https://www.pewresearch.org/politics/2020/06/25/republicans-democrats-move-even-further-apart-in-coronavirus-concerns/ (accessed June 25, 2021).

[B31] Rentfrow P. J. Gosling S. D. (2003). The do re mi's of everyday life: the structure and personality correlates of music preferences. J. Pers. Soc. Psychol. 84, 1236–1256 10.1037/0022-3514.84.6.1236 12793587

[B32] Rentfrow P. J. Gosling S. D. Potter J. (2008). A theory of the emergence, persistence, and expression of geographic variation in psychological characteristics. Perspect. Psychol. Sci. 3, 339–369. 10.1111/j.1745-6924.2008.00084.x 26158954

[B33] Rentfrow P. J. Jost J. T. Gosling S. D. Potter J. (2009). Statewide differences in personality predict voting patterns in 1996-2004 U.S. presidential elections, in Series in Political Psychology. Social and Psychological Bases of Ideology and System Justification, eds Jost J. T. Kay A. C. Thorisdottir H. (Oxford: Oxford University Press), 314–347.

[B34] Robinson W.S. (1950). Ecological correlations and the behavior of individuals. Am. Sociol. Rev. 15, 351–357. 10.2307/2087176

[B35] Royston P. (1993). A Toolkit for testing for non-normality in complete and censored samples. J. R. Stat. Soc. Series D 42, 37–43. 10.2307/2348109

[B36] Shook N. Sevi B. Lee J. Fitzgerald H. N. Oosterhoff B. (2020). Who's listening? predictors of concern about COVID-19 and preventative health behaviors. PsyAiXiv. 10.31234/osf.io/c9rfg

[B37] Terracciano A. Costa Jr P. T. (2004). Smoking and the five-factor model of personality. Addiction 99, 472–481. 10.1111/j.1360-0443.2004.00687.x 15049747 PMC2376761

[B38] U.S. Census Bureau (2020). State of Residence by Place of Birth – ACS Tables. Available online at: https://www.census.gov/data/tables/time-series/demo/geographic-mobility/state-of-residence-place-of-birth-acs.html (accessed February 22, 2021).

[B39] U.S. Census Bureau (2021a). Hispanic or Latino by Race (American Community Survey, 2019 Five Years Estimates). Available online at: https://data.census.gov/cedsci/table?g=0100000US.04000.001&tid=ACSDT5Y2019.B03002 (accessed August 8, 2021).

[B40] U.S. Census Bureau (2021b). American Community Survey. ACS 5-Year Educational Attainment Estimates (2013–2017). Available online at: https://data.census.gov/cedsci/table?q=S1501&tid=ACSST1Y2019.S1501 (assessed February 1, 2021).

[B41] U.S. Census Bureau, Population Division (2020). Annual Estimates of the Resident Population for Counties in the United States: April 1, 2010 to July 1, 2019 (CO-EST2019-ANNRES). Available online at: https://www.census.gov/data/datasets/time-series/demo/popest/2010s-counties-total.html

[B42] United States Presidential Election (2021, May 15). In Wikipedia. https://en.wikipedia.org/w/index.php?title=1996_United_States_presidential_election&oldid=1036874664

[B43] Webb Hooper M. Nápoles A. M. Pérez-Stable E. J. (2020). COVID-19 and racial/ethnic disparities. JAMA 323, 2466–2467. 10.1001/jama.2020.8598 32391864 PMC9310097

[B44] Wood D. Rogers K. H. (2011). Regional differences in personality exist, but how do we get to them? The case of conscientiousness. Am. Psychol. 66, 917–918. 10.1037/a0024719 22121993

[B45] Zientek L. R. Thompson B. (2006). Commonality analysis: partitioning variance to facilitate better understanding of data. J. Early Interv. 28, 299–307. 10.1177/105381510602800405

[B46] Zvolensky M. J. Taha F. Bono A. Goodwin R. D. (2015). Big five personality factors and cigarette smoking: a 10-year study among US adults. J. Psychiatr. Res. 63, 91–96. 10.1016/j.jpsychires.2015.02.008 25799395 PMC4422054

