# Supplementary material for: Personality and Its Partisan Political Correlates Predict U.S. State Differences in Covid-19 Policies and Mask Wearing Percentages
Source: Front Psychol. 2021 Sep 27;12:729774. doi: 10.3389/fpsyg.2021.729774 (PMC8502796; doi:10.3389/fpsyg.2021.729774)
Supplement: Supplementary file 1 [file Data_Sheet_1.docx]

Supplementary Material

Table S1: State-level correlations for *Conscientiousness* and *Openness* with percentage of state residents who smoke and the percentage of votes for Democratic presidential candidates in U. S states in the past six national elections.

|  | % of State Residents who Smoke, 2009*^a^* | Dem Pres Candidate 1996 *^b^* | Dem Pres Candidate 2000 | Dem Pres Candidate  2004 | Dem Pres  Candidate  2008 | Dem Pres Candidate 2012 | Dem Pres Candidate  2016 | Dem Pres Candidate 2020 |
| --- | --- | --- | --- | --- | --- | --- | --- | --- |
| Conscientiousness | 0.17 | -0.41** | -0.35* | -0.43** | -0.36* | -0.42** | -0.33* | -0.34* |
| Openness | -0.39* | 0.44** | 0.47*** | 0.53*** | 0.44** | 0.46*** | 0.57*** | 0.58*** |
| *^a^* CDC, 2010  *^b^* 1996 United States Presidential Election | | | | | | | | |

* <= .05

** <= .005

*** <= .0005

Table S2: The beta coefficients for regression analyses of state differences in the stringency of Covid-19 restrictions as a function of the number and nature of the other predictors (the “regression context”).

| Predictor  & Number of Predictors | 4 | 5 | 6 | 7 | 8 | 9 |
| --- | --- | --- | --- | --- | --- | --- |
| Openness Rank | 0.061 | 0.050 | 0.057 | 0.069 | 0.082 | 0.081 |
| Conscientious-  ness rank | -0.213 | -0.188 | -0.181 | -0.194 | -0.203 | -0.196 |
| % Democratic state legislators | 0.677 | 0.639 | 0.666 | 0.644 | 0.637 | 0.632 |
| % Born out of state | -0.094 | -0.097 | -0.090 | -0.098 | -0.088 | -0.085 |
| Cases/100k |  | -0.098 | -0.080 | -0.078 | -0.083 | -0.088 |
| Urbanization |  |  | -0.041 | -0.070 | -0.061 | -0.045 |
| % of not White state residents |  |  |  | 0.058 | 0.06 | 0.037 |
| % of college graduates |  |  |  |  | -.061 | -0.066 |
| Median purchasing power |  |  |  |  |  | -0.032 |

Table S3: The beta coefficients for regression analyses of state differences in mask wearing as a function of the number and nature of the other predictors (the “regression context”).

| Predictor  & Number of Predictors | 4 | 5 | 6 | 7 | 8 | 9 |
| --- | --- | --- | --- | --- | --- | --- |
| Openness Rank | 0.200 | 0.189 | 0.125 | 0.159 | 0.159 | 0.162 |
| Conscientious-  ness rank | -0.013 | 0.004 | -0.003 | -0.024 | -0.024 | -0.042 |
| % Democratic state legislators | 0.587 | 0.544 | 0.457 | 0.439 | 0.439 | 0.451 |
| % Not White state population | 0.262 | 0.276 | 0.176 | 0.184 | 0.184 | 0.238 |
| Cases/100k |  | -0.085 | -0.168 | -0.183 | -0.183 | -0.171 |
| Urbanization |  |  | 0.232 | 0.264 | 0.264 | 0.236 |
| % College graduates |  |  |  | -0.137 | -0.137 | -0.126 |
| % Born out of state |  |  |  |  | -0.001 | -0.008 |
| Median purchasing power |  |  |  |  |  | 0.078 |

**REFERENCES**

Centers for Disease Control and Prevention. (2010). State-Specific Prevalence of Cigarette Smoking and Smokeless Tobacco Use Among Adults --- United States, 2009 *Morbidity and Mortality Weekly Reports,*  59(43);1400-1406. Available at https://www.cdc.gov/mmwr/preview/mmwrhtml/mm5943a2.htm (accessed June 8, 2021).
